# The Networked Data Lab: using linked data to understand intermediate care services in the United Kingdom

**DOI:** 10.23889/ijpds.v9i5.2834

**Published:** 2024-09-18

**Authors:** Caroline Fraser, Tom Prendergast, Chamut Kifetew, Zoe Ruziczka, Hannah Knight

**Affiliations:** 1 The Health Foundation

Intermediate care (IC) is short-term care aimed at maximising patients’ independence. Across the UK, scaling up IC is included in post-pandemic recovery plans. This could reduce pressures on acute services through timely discharge or preventing admissions. The issues IC seeks to address are common across high-income countries.

The Networked Data Lab is a network of analytical teams embedded in the UK health and care system. Each year, we support five local partners with acquisition, linkage and analysis of health and social care datasets. Our 2023-24 project is focused on IC.

Patients and the public (PP) played an integral role in throughout our project. We established a panel of individuals with lived experience of IC to assist with the research questions, interpretation, and communication of results. Our partners all consult PP in their areas to inform their analyses.

Our partners’ analyses of linked patient-level data on IC will provide information about who is receiving IC, changes over time and outcomes for patients. We are also analysing national linked data for England on intermediate care following hospital discharge.

Preliminary results include:

The age and health needs of people receiving bed-based intermediate care has increased over time (Grampian, Scotland)
Intermediate care following a hospital admission is more common for emergency admissions than elective admissions (Leeds, England)
Large variation in intermediate care delivery between neighbouring boroughs (North West London, England)

Full results are expected in April 2024.

Our work highlights the importance of linking data to answer important health and care questions.

